# Cardiovascular PIEZO discovery continues apace to reveal myocardial nerves feeling posture and blood loss

**DOI:** 10.1038/s44161-026-00802-w

**Published:** 2026-03-24

**Authors:** David J. Beech

**Affiliations:** School of Medicine, LIGHT Building, Clarendon Way, University of Leeds, Leeds LS2 9JT, UK

## Abstract

The idea of myocardial mechanical reflexes was previously considered but it struggled to gain traction in mainstream thinking. New research shows PIEZO2 ion channels conferring vagal force sensing in atrial and ventricular myocardium to protect the circulation as blood volume changes with gravity and injury.

As beautifully illustrated by Leonardo da Vinci over 500 years ago, the heart is a complex mechanically vibrant organ with structures that reflect its functions. It exists in a mechanically vibrant whole-body with which it interacts on immediate and life course time scales. While many studies characterized and sought to explain or modify this cardiovascular mechanobiology in health and disease, progress was hampered in important regards by uncertainty about the molecular basis of the mechanical force sensors and a view that force sensing may be mediated by a synergy of molecular components rather than an individual sensor. The perspective began to change with patch-clamp and RNA interference studies of cultured neuroblastoma cells that revealed the PIEZO1 and PIEZO2 channels ^1^ and then observation of a critical role of PIEZO1 in endothelial fluid flow sensing and the determination of vascular architecture ^2^. We now have overwhelming evidence and agreement amongst investigators that PIEZO channels are highly sensitive tuneable force sensors dedicated to force detection and transduction through their unique physical attributes (Figure 1) ^3^. With this advance came powerful new ways to test ideas about cardiovascular mechanobiology.

Each PIEZO protein is embedded in the plasma membrane or sometimes another membrane via 38 membrane-spanning helices ^3^. Each channel comprises three PIEZO1s or three PIEZO2s integrated as PIEZO1 or PIEZO2 channels with 114 (i.e., 3×38) membrane-spanning helices. Most of these helices contribute unusual blade-like structures that radiate laterally from the central ion conduction pathway (Figure 1A, B). When the channels are closed in the absence of force, they create an inverted dome with the membrane pushed inwards (Figure 1C). With force, there is flattening as the blades spread out, triggering non-selective cation conduction and cell excitation (Figure 1D).

It has been appreciated for decades that the heart monitors and adapts itself partly through embedded neuronal reflexes for coordinated cardiac and whole-body adjustments ^4^. Experiments to investigate this biology were challenging, however, because it was difficult to definitively apply local mechanical stimuli to important parts of the heart. While such studies supported ideas about myocardial mechanical reflexes and their independence from other reflexes (e.g., the baroreceptor reflex), there was unclear or apparent minor significance of the observed phenomena ^4^. With the discovery of PIEZOs ^1^, the vast expansion of PIEZO knowledge that followed ^3^ and new more sophisticated experimental techniques, it became timely to look again. In their elegant Article in *Nature*, Liu et al show us what has now been possible, 25 years later ^5^. They began with the premise of there being incompletely understood neuronal surveillance of cardiovascular activity and went on to reveal important new information about mechanical detection in the heart and roles of this detection in how upright stance is usually achieved without the individual fainting and how problems of postural (orthostatic) hypotension and cardiovascular trauma may arise.

Liu et al ^5^ adapted human tilt table techniques to study anaesthetized mice with 180 degrees rotation during monitoring of cardiovascular parameters. They found an upright position of the mouse caused a drop in arterial pressure that was compensated by increased heart rate unless there was cervical vagotomy or vagal nerve transection below the superior laryngeal nerve, which left the baroreceptor reflex intact. Gene- and promoter-specific manipulations were used to test ideas about the molecules contributing. PIEZO2 emerged as crucial. With *Piezo2* gene disrupted in vagal neurones, the tilt table effect was impaired while the baroreceptor reflex was normal. Injection of vagal neurones with a reporter enabled elegant neuronal mapping, revealing dense PIEZO2-containing innervation across cardiac compartments with characteristic end-net (bead-like) nerve endings. Diphtheria toxin-mediated ablation of the PIEZO2-containing vagal neurones prevented the tilt table response. Vagal electrophysiological recording below the superior laryngeal nerve showed PIEZO2-dependent firing timed with the electrocardiogram, suggesting beat-to-beat mechanical force sensing. When PIEZO2 neurones were specifically stimulated using optogenetics, there were reductions in blood pressure and heart rate.

Liu et al ^5^ went on to explore the relevance to changes in blood volume, reasoning the force sensing enables the heart to feel what is happening elsewhere in the body. They observed these PIEZO2 channels being responsible for decreased vagal activity when circulating blood was withdrawn and increased activity when circulating volume was increased, suggesting the PIEZO2 neurones inform the heart about circulating volume. Acute trauma was studied by tail-bleeding that caused the mice to lose up to a quarter of their blood volume in 30 minutes and larger volumes over longer periods. Mice with PIEZO2 deleted in vagal neurons struggled to generate a compensatory increase in heart rate and maintain blood pressure and their survival with prolonged bleeding was reduced. Blood loss from the jugular vein was similarly less well compensated compared with control wild-type PIEZO2 mice. Therefore, through powerful PIEZO targeting and complementary in vivo and genetic techniques, important mechanical force sensing in the heart has been recognised and its significance explained.

While these findings are new and exciting, they are not the first time we have seen PIEZO2 acting in and around the heart ^6^ (Figure 2). PIEZO2 was also seen in the baroreceptor reflex, with PIEZO2-containing nerve terminals forming claw-like structures along the outer edge of the aortic arch, positioning the channels ideally to detect pressure pulses. PIEZO2 was seen in aortic valve and coronary vascular formations. PIEZO2-deleted mice exhibited cardiac hypertrophy and increased left ventricular and interventricular septal thicknesses, suggesting physiological roles in protecting against myopathy. In disease it may be different, with PIEZO2 driving adverse cardiac adaptations. Recapitulation of a human PIEZO2 gain-of-function variant in mice increased heart weight and reduced heart length. PIEZO1, which did not contribute to the mechanical reflex in the Liu et al studies ^5^, was also important in the heart and perhaps more so in some situations such as pathological cardiac stress, with striking increases in PIEZO1 expression and function in cardiac myocytes in animal models of human heart failure ^6^. PIEZO1 contributed to the baroreceptor reflex in concert with PIEZO2. Why two PIEZOs should sometimes be used and PIEZO2 alone favoured in some situations, as in Liu et al ^5^, is not entirely clear but PIEZO2 channels differ from PIEZO1 channels in the types of mechanical forces to which they respond and the kinetics of their responses ^3^, properties that may be advantageous in nerve terminals.

Capability to manipulate genes specifically in subsets of cardiac nerves was a challenge addressed admirably by Liu et al ^5^ through multiple independent technical approaches, giving confidence in the myocardial nature of the neuronal sensing. We can nevertheless expect future studies to advance the knowledge of specific gene expression patterns in cardiac nerve types, enabling even more refined targeting for additional rigorous testing and the generation of new knowledge about important subtleties in these still quite poorly understood myocardial neuronal surveillance mechanisms. There is also the challenge of understanding how decreased force from reduced blood volume could be sensed by a channel that is activated by force. The vagal PIEZO2 channels seem to exist at an optimum point of partial activation, such that reduced force decreased the channel activity and enhanced force increased it. This would be a logical explanation; the channel activity was not measured, however, and it is unclear how the channels ensure a ‘Goldilocks’ mechanical setpoint.

While Liu et al ^5^ show excellent science and new knowledge, it is valuable to see it in the wider context of the many additional PIEZO discoveries ^3^. A myriad PIEZO roles are already appreciated in cardiovascular biology after only a decade and half of research (Figure 2) and there are many other roles known that are beyond or integrated with cardiovascular biology such as in the respiratory and immune systems^3^. Both PIEZOs are widely expressed (PIEZO1 most of all) ^3^ and both may be thought of as plug-in force sensing machines serving diverse cellular needs, adapted and tuned to different cell types through many mechanisms including splice variation and differential lipid and protein regulation ^3,7,8^.

Despite the attention it attracted so far, PIEZO research almost certainly still has plenty more to yield by revealing much-needed understanding of how the channels work in diverse cell types and context and how they are regulated and integrate and signal. There is also more to know about how PIEZOs relate to human conditions through pathological *PIEZO* variations and, probably more commonly, PIEZO modulations in disease and ageing as cell and tissue mechanics alter (e.g., through stiffening and scarring) and PIEZO channel functions are altered by dysregulations of lipids and other factors. Artificial PIEZO modulation in therapeutics is also expected to come to the fore, as seen recently with cardio protection in cancer therapy ^9^.

## Figures and Tables

**Figure 1:**
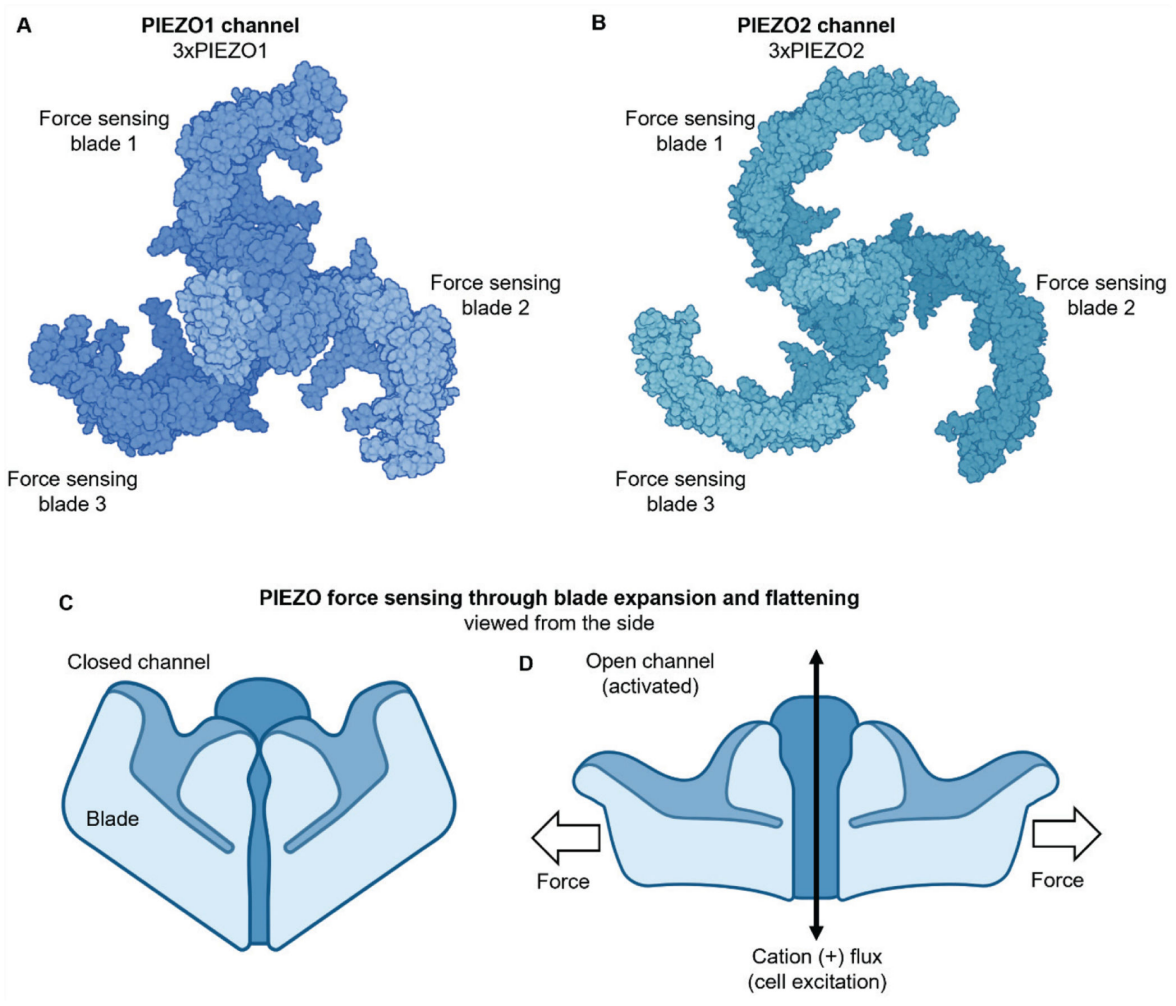
Uniqueness of PIEZO force sensors and their blades. **A, B**. Top (helicopter) views of PIEZO1 (**A**) and PIEZO2 (**B**) channels based on Protein Data Bank structures. **C, D**. Side view sketches of PIEZO channel in closed non-conducting (**C**) and open conducting (**D**) states, emphasizing the importance of blade structures in sensing force through structural rearrangement in response to force. Opening of the channel leads to cation (Ca^2+^, Na^+^, K^+^) conduction that excites the cell. Lipid membranes are important for PIEZOs but not illustrated here. Created with templates from BioRender.com.

**Figure 2:**
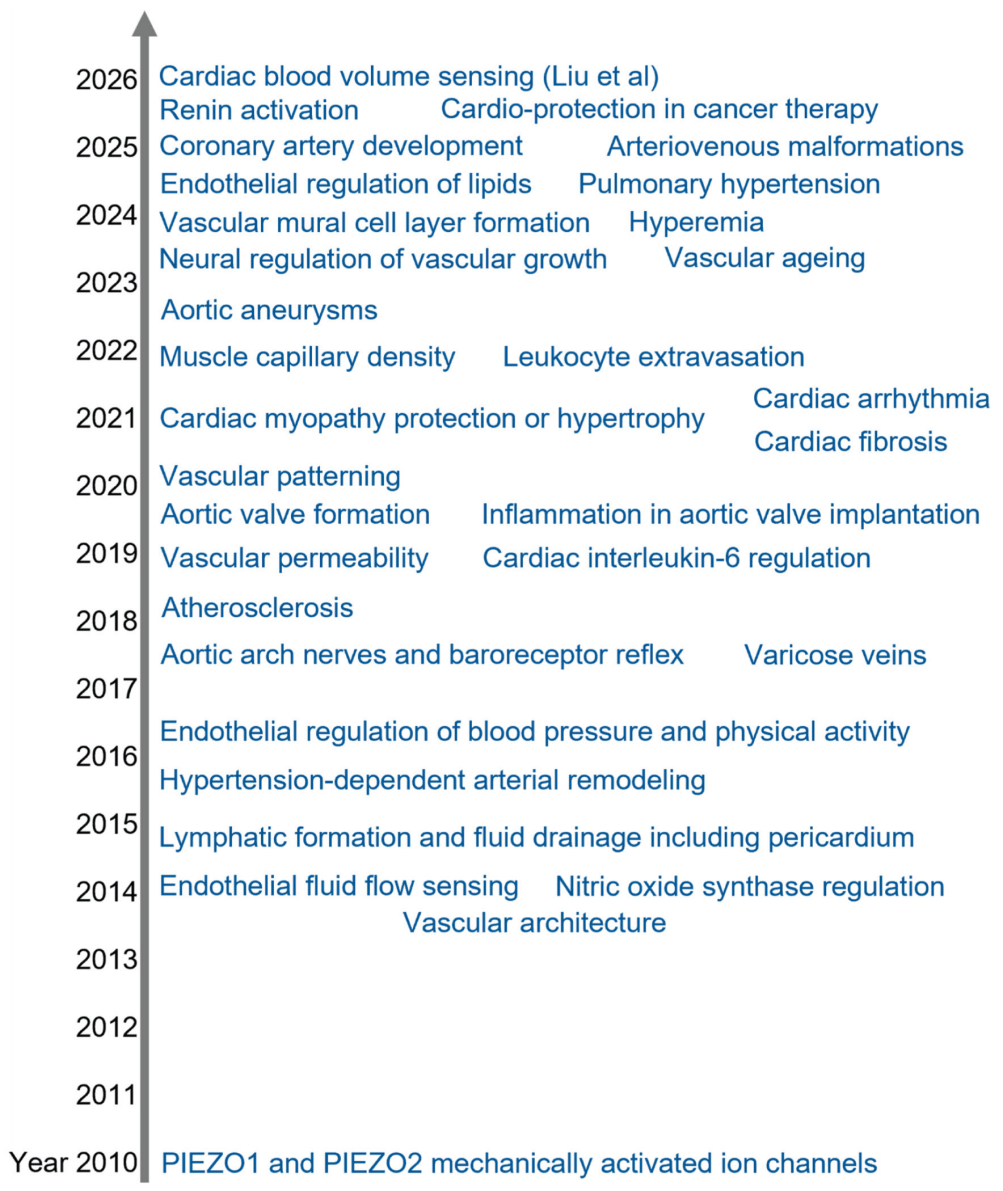
Expanding knowledge of PIEZOs in cardiovascular biology. A timeline from year 2010 to the present, showing important discovery milestones according to their publication date. Further information and study details are reviewed elsewhere ^3,6,10^ or are available in recent publications since these reviews.
